# Modern Medical Therapeutics

**Published:** 1878-03

**Authors:** 


### Books Reviewed.

*Modern Medical Therapeutics: A Compend of Recent Formulæ and Specific Therapeutical Directions, from the Practice of Eminent contemporary Physicians, American and Foreign.* By GEO. H. NAPHEYS, A. M., M. D. Fifth Edition Enlarged and Revised.

*Modern Surgical Therapeutics: A Compend of Current Formulæ, Approved Dressings and Specific Methods for Treatment of Surgical Diseases and Injuries.* By GEO. H. NAPHEYS, A. M., M.D. Revised to the most recent Date. Philadelphia: D. G. Brinton, 115 South Seventh St. 1878. Price per volume in cloth $4.00.

The former of these volumes has a reputation derived from a number of years use, while the companion volume now appears in its first edition. They are such volumes as the busy practitioner will prize most highly, as nothing but practical matter, brief, specific, and well-approved is found in them. They constitute a compend of practice in medicine and surgery, wisely made up from the best and most recent authorities. Separate sections are devoted to the different diseases and the matter which is mainly in the shape of formulæ, copied literally from the practice of various physicians named, is ample enough to cover the ground well and allow quite an opportunity of selection from varying methods and means. The previous edition of the volume on Medical Therapeutics was issued only a short time since and in this last revision the work attains the size of an octavo of nearly six hundred pages, with which the Surgical volume matches. It may be known to our readers that Dr. Napheys died before completing his preparation of the latter volume and that this labor was undertaken and completed by the editor and publisher Dr. D. G. Brinton of Philadelphia well known as the editor of the *Half Yearly Compendium of Medical Sciences.* It is sufficient to say that the work has been judiciously and thoroughly done; we recommend others to judge of the volumes for themselves. W.

